# Complementary and alternative medicine (CAM) providers’ views of chronic low back pain patients’ expectations of CAM therapies: a qualitative study

**DOI:** 10.1186/1472-6882-12-234

**Published:** 2012-11-27

**Authors:** Lisa M Schafer, Clarissa Hsu, Emery Rose Eaves, Cheryl Ritenbaugh, Judith Turner, Daniel C Cherkin, Colette Sims, Karen J Sherman

**Affiliations:** 1 Center for Community Health and Evaluation, Group Health Research Institute, 1730 Minor Avenue, Suite 1600, Seattle, WA, 98101, USA; 2 Department of Family and Community Medicine, University of Arizona, 1450 N Cherry Avenue, Tucson, AZ, 85719, USA; 3 Department of Psychiatry and Behavioral Sciences, University of Washington, Box 356560, Seattle, WA, 98195-6560, USA; 4 Group Health Research Institute, 1730 Minor Avenue, Suite 1600, Seattle, WA, 98101, USA

**Keywords:** Expectations, Providers, Qualitative research, Chiropractic, Massage, Acupuncture, Yoga, Complementary medicine, Alternative medicine

## Abstract

**Background:**

Some researchers think that patients with higher expectations for CAM therapies experience better outcomes and that enthusiastic providers can enhance treatment outcomes. This is in contrast to evidence suggesting conventional medical providers often reorient patient expectations to better match what providers believe to be realistic. However, there is a paucity of research on CAM providers’ views of their patients’ expectations regarding CAM therapy and the role of these expectations in patient outcomes.

**Methods:**

To better understand how CAM providers view and respond to their patients’ expectations of a particular therapy, we conducted 32 semi-structured, qualitative interviews with acupuncturists, chiropractors, massage therapists and yoga instructors identified through convenience sampling. Interviews were recorded, transcribed and analyzed thematically using Atlas ti version 6.1.

**Results:**

CAM providers reported that they attempt to ensure that their patients’ expectations are realistic. Providers indicated they manage their patients’ expectations in a number of domains— roles and responsibilities of providers and patients, treatment outcomes, timeframe for improvement, and treatment experience. Providers reported that patients’ expectations change over time and that they need to continually manage these expectations to enhance patient engagement and satisfaction with treatment.

**Conclusions:**

Providers of four types of CAM therapies viewed patients’ expectations as an important component of their experiences with CAM therapy and indicated that they try to align patient expectations with reality. These findings suggest that CAM providers are similar in this respect to conventional medical providers.

## Background

Patient expectations are part of the contextual background that may contribute to therapeutic improvement. Treatment providers are known to play a role shaping patient expectations of a treatment [1-3], which can lead to improved treatment outcomes [2,4,5]. Some researchers think that patients’ positive expectations of treatment, amplified by provider optimism and enthusiasm, are largely responsible for positive outcomes reported by patients who use complementary and alternative medicine (CAM) for management of bothersome symptoms, including pain [6-10].

In some clinical trials, high patient pre-treatment expectations of CAM therapies predicted better treatment outcomes [11-15]. However, not all studies have found this relationship [16] and one study reported a negative association [17]. Studies of associations between patient pre-treatment expectations of conventional medical treatments and patient outcomes have also been inconsistent [18-20].

Patient expectations of a therapy may change after they have started the treatment, and conceivably, treatment outcomes are influenced more by patients’ expectations after the therapy has begun than by pretreatment attitudes. Providers may play important roles in this process by purposefully or inadvertently shaping their patients’ expectations [3,21,22]. Several studies have found that providers often reorient patient expectations to better match what providers believe to be realistic [23-26]. For example, before a surgical procedure some providers educate patients about the process and possible outcomes in terms of timeframes for changes, level of pain reduction, and pain medicine requirements [27-29]. Such management of patients’ expectations has been shown to be associated with increased patient satisfaction, enhanced patient engagement in treatment, and improved quality of care [23-26]. Other research suggests there is a dynamic interaction between patients’ expectations and their experiences with treatment [30,31]. These findings illustrate the importance of increased understanding of the concept of patient expectations related to a treatment and how they may be influenced by providers.

Towards this objective, as part of a study to develop a validated questionnaire for assessing expectations of patients initiating CAM therapies for chronic back pain, we interviewed experienced CAM providers concerning their perceptions of what patient expectations are when they seek CAM therapy and how they attempt to manage these expectations. We chose to focus on chronic back pain because it is one of the most common reasons for which CAM care is used, is a very common problem lacking highly effective treatments, and the non-specific effects of treatments, including patient expectations, are likely to be larger for the non-pharmacological therapies we focused on [32,33]. We chose chiropractic, massage, acupuncture and yoga because they are among the most common CAM therapies used for back pain [34].

We use the term “patient” throughout this article to refer to any person seeking relief from back pain by making visits for acupuncture, chiropractic, or massage therapies or by attending yoga classes under the supervision of a yoga instructor. “CAM providers” refers to individuals who deliver acupuncture, chiropractic, or massage therapy, or teach yoga.

## Methods

### Study sample and data collection

The study team developed a conceptual model of patient expectations based on a review of the relevant literature and prior research experience (see Additional file 1). This model informed the contents of our interview guide. In addition to asking providers about their approach to treating patients with back pain, interview questions solicited providers’ perspectives on types of patient expectations and the extent to which they identified or responded to those expectations. The questions were:

General expectations

•Briefly walk me through how you would go about assessing and working with a patient/client with chronic low back pain?

•I would like to talk about the types of expectations your patients/clients with chronic low back pain tend to have. What, if any, expectations do you find they have?

•What are some examples of how patients/clients talk about their expectations?

•What are other ways that patients/clients communicate their expectations?

•How do patients/clients’ expectations change over time?

•What kinds of things are patients/clients surprised by during the course of their experience with you?

•What kinds of things are you surprised by when working with patients/clients with chronic low back pain?

Assessing and managing expectations

•To what extent do you consciously try to manage or shape the expectations of patients/clients with low back pain?

•Do you make an effort to formally or systematically assess your patients’/clients’ expectations regarding treatment? If so, how? If not, do you have indirect ways of assessing expectations?

•Can you describe some situations where you have had to manage a patient/client’s expectations? What strategies do you have for doing this?

The study used a convenience sample that included providers from the three most commonly used CAM therapies for back pain (chiropractic, acupuncture, and massage therapy), as well as yoga, which is growing in popularity. Based on our extensive knowledge of providers of these CAM therapies, the study team identified providers with reputations for providing high-quality care to individuals with chronic low back pain. Study team members invited these providers to participate in the study through an initial mailing followed by a phone call. We invited 41 providers to participate, and 33 were interviewed. We pre-tested the interview guide with one provider of each type in Seattle, which resulted in minor revisions in the order of the questions to improve the flow of the interviews.

Semi-structured, in-depth interviews were conducted with 10 acupuncturists, seven chiropractors, nine massage therapists, and eight therapeutically-oriented yoga instructors (total of 33 interviews) in Tucson, AZ and Seattle, WA in May-August 2010. Eleven interviews were completed in Tucson and 22 in Seattle. Three experienced interviewers (LMS, CH, ERE) conducted the interviews. All were done in person at a private location convenient to the provider and lasted approximately an hour. Providers were compensated $100 for their time. Interviews were recorded and transcribed for analysis.

Because this research did not solicit personal health information, it was exempted from institutional review board (IRB) approval, according to the Human Subjects Review Committee of the Group Health Research Institute in Seattle. In Tucson, the study used a modified consenting process and received IRB approval from the University of Arizona’s Human Subjects Review Committee.

### Data analysis

One study team member (KJS) read all of the transcripts and four team members read all transcripts for a specific type of CAM. Team members elucidated major themes to inform subsequent data analysis.

Interview transcripts were analyzed thematically by the three interviewers using an immersion/crystallization approach, which emphasizes gaining an in-depth knowledge of the data in order to identify key themes [35]. One interviewer (CH) drafted an initial coding scheme based on the conceptual model, team discussions of findings, and initial impressions from the data. Other study team members reviewed the draft coding scheme and made revisions. All interviewers coded six transcripts, which were then compared and discussed to reconcile differences and ensure reliability among coders. Additional revisions to the coding scheme were made based on the initial coding. The final coding scheme was reviewed and approved by the full study team. One interviewer in Seattle (LMS) coded the remaining 18 transcripts for interviews conducted in Seattle and one interviewer in Tucson (ERE) coded the remaining eight transcripts for interviews conducted in Tucson. The coding scheme was further developed through an iterative process that included minor changes and additions. Provider comments reflecting more than one theme were assigned multiple codes. Coding of transcripts was managed in the qualitative analysis software Atlas.ti.

Interviews resulted in a complex data set that included information concerning the types and potential effects of patients’ expectations, changes in expectations over time, and the distinction between expectation and hope. This qualitative database was analyzed for themes and an assessment was made of whether certain themes were more prevalent for some CAM therapies than for others. Additional levels of coding were completed on data within the code “provider management of expectations” to identify the subthemes that were emerging from the data in this specific area. Prevalent subthemes were summarized in the form of a coding memo. The entire study team discussed the coding memo to guide additional analysis and reach consensus on findings. Once these themes were discussed and agreed upon by the team they were used to construct the findings section of this paper.

## Results

A total of 33 providers completed interviews. One provider in Seattle practiced both massage and yoga therapies (Table 1). A number of themes emerged related to CAM patients’ expectations and providers’ management of those expectations.

**Table 1:** Characteristics of CAM providers interviewed

	**Acupuncturists**	**Chiropractors**	**Massage therapist**	**Yoga instructor**	**Totals**
*Interview location*					
Seattle	6	5	6*	6*	23
Tucson	4	2	3	2	11
*Years practicing CAM*					
Less than 5	0	0	1	0	1
5-9	3	1	2	1	7
10-20	6	0	4	1	11
More than 20	2	6	2	5	15
*Gender*					
Female	5	1	8	8	22
Male	5	6	1	0	12

### Provider management of patient expectations

Providers reported a strong belief in the importance of assessing and managing patient expectations. The specific domains where providers indicated they managed expectations included roles and responsibilities of providers and patients, treatment outcomes, timeframe for improvement, and treatment experience. Despite the variability in the CAM therapies studied, findings related to patient expectations were remarkably similar across the four CAM provider types. Any differences between different types of providers are explicitly noted. The following themes resulted from our analysis of providers’ statements related to the management of patient expectations and each them is discussed under a separate subheading.

#### *Process of patient expectation management*

CAM providers acknowledged managing patient expectations to some extent through various formal and informal processes. However, they often struggled to articulate the various ways they managed expectations. Only through guided probing over the course of the interview did the depth and complexity of their understanding and response to this concept become apparent. For example, when asked explicitly if they manage expectations, three providers initially indicated they did not, but then later provided examples of managing expectations.

Interviewer: “*And to what extent do you consciously try to manage or shape your patients’ expectations?*

Respondent: “*I don’t. Not at all. Because…I think their expectations are based upon their lifestyles and personal needs. How can I change their personal needs?*

Later in the interview, the same chiropractor explained,

“*I think my job is to identify their problem, tell them honestly what I can do for them, teach them the best I can…to help manage this problem. And then only, when it comes to their expectations, only correct them when I think they’re wrong.” –* Chiropractor 1, Seattle

Overall, providers said it was important to attempt to align patient expectations with realistic or likely outcomes in order to improve patient engagement and satisfaction. When asked about the extent to which she manages expectations, one provider responded,

*“Quite a bit. I mean, we talk about it. We put it out there. I’ll want to know what they want, what they expect to get, and then I’ll talk to them about more realistic expectations and whether they jibe and if they’re comfortable with that because I don’t want to progress with treatment if somebody has unrealistic expectations that could really disappoint them in the end and then misrepresent the profession. Misrepresent the efficacy of massage or of yoga therapy. So, I want them to be very well informed about how and what. What we can look for as signs of success that may be different than what they expect.”* – Massage therapist and yoga instructor 2, Seattle

Generally, providers said that they did not have a mechanism for formally assessing patient expectations, but they intentionally integrated systems and processes for managing expectations into the way they practiced (e.g., through intake forms or procedures, providers’ approaches to providing care). Most providers begin explicitly managing patient expectations early on in care, generally at the first visit, but in some cases in the initial phone call. Providers often framed management of patient expectations as “patient education” and considered it part of their role as a CAM provider. One provider said,

*“And so that’s the practitioner’s job, to really get firm on what [the patients’] expectations can be….So really set the tone from the get-go of what the expectation should be rather than missing that and leaving it up to the patient to have their own expectations.”* – Acupuncturist 3, Seattle

#### *Expectations regarding patient and provider roles and responsibilities*

CAM providers spoke about patients expecting the providers to “fix” their chronic low back pain and the providers’ need to mitigate unrealistic expectations. Some providers stated that some patients saw them as “miracle workers” and that they work to diffuse this myth and clarify their roles as health care providers. Overwhelmingly, providers indicated they thought that, for optimal results, patients must take an active role in their healing process. Providers said that they tailor messages and provide education in an effort to manage patients’ expectations and facilitate patient engagement and confidence in their own ability to manage their pain. One acupuncturist explained,

*“I talk to them a lot and I educate them a lot about what I’m doing and why…. I think sometimes people are so used to going to a medical appointment and looking for a solution and imagining that it’s kind of like an oil change. That they’re going to go in and lay on a table, somebody’s going to stick needles in them, and that’s it. That’s all they have to do. So, when I talk to them about being self-responsible and looking at their lifestyle, and looking at their feelings and looking at their habits, you know, as well as their exercise and you know, other things. It’s a lot—people are often surprised. ‘Wow. Like you’re asking me to really change.’* – Acupuncturist 4, Tucson

Providers indicated that a “fix me” expectation signals that the patient is disengaged from his/her body and does not recognize his/her own role in the healing process. They emphasized that patients with chronic low back pain likely need to change lifestyle habits and engage in self-care activities to achieve sustained pain relief. All four types of providers said they engage patients in dialog throughout the therapy about this and are explicit with patients about their responsibilities. According to one provider,

*“And the other expectation, if somebody comes to see me and they’re telling me that, ‘You’re the only guy that can help me and nobody else has listened to me’ then I think that there are some unrealistic expectations going on there. And they’re investing too much in me and not enough in themselves. And I’ll try and tell them that we’ll do some stuff in the office here but you’re going to need to do some stuff on your own.” –* Acupuncturist 5, Seattle

#### *Treatment outcome expectations*

Overall, providers said the primary expectations of patients with chronic low back pain were decreased pain and, as a component of that, improved function. These expectations aligned with providers’ goals for treating patients; however, providers agreed that patient characteristics such as physiology, diagnosis and age can affect treatment outcomes. Providers reported that in many cases, treatment is unlikely to cure a patient’s chronic low back pain, but it can be used effectively as a tool to manage the condition. As a result, providers said they educate patients regarding what is and is not realistic to expect from the treatment, both in terms of the amount of pain relief they can expect and the extent to which treatment will restore their ability to engage in various activities. They also noted that effectively managing these expectations is challenging because of the individual variability among patients and the complexities of the healing process. One provider said,

*“I would say we very consciously shape what the expectation is. So we obviously say we certainly would love to reduce your chronic pain. That would be great. But that’s the one thing we don’t know we can do. That’s so personal. But what we do know is that we can give you a sense of hope, a sense of optimism, some new tools that you can use. A place where you’re totally understood and accepted. So we also list out what we are really sure we can deliver.”* – Yoga instructor 6, Seattle

Providers said patient expectations concerning treatment outcomes often change over time, which continually influences their approach to patient education and how they respond and manage expectations. Overall, providers said they have a holistic approach to care and that it is not uncommon for patients to experience positive outcomes other than pain relief (e.g., improved sleep, better stress management, increased energy) that they associate with treatment. As patients become more comfortable with the provider and treatment, and experience positive outcomes, their expectations may shift to expecting more from the treatment. Providers reported these new expectations might be unrealistic and said that they then manage these expectations by reminding patients of the progress they have made, and by providing education concerning when and if the treatment is appropriate for a particular condition. This was particularly true for massage therapists. One massage therapist said,

*“Because I make it a point to point out their progress their expectations start to change as they see themselves changing. And a lot of it isn’t so much that the pain’s gone away, their function is increasing within that pain…And once they start to see that, see their function changing, they start to have hope, and they start to expect themselves to do more. And they start to buy into the program and be more of an active participant.”* – Massage therapist 7, Tucson

#### *Expectations regarding timeframe for improvement*

Providers agreed that many patients with chronic low back pain want to know early on in treatment when they can expect pain relief, even if they have preconceived notions about how long that should take. Providers said that chronic low back pain is often a complex and multifaceted condition that has various contributing factors (e.g., physiology, history of injury, nutrition, patient’s age, patient’s lifestyle and habits) that take time to identify, untangle and address.

Because of this complexity, providers said that they are careful to explain to patients that it will likely be multiple sessions before they notice progress or change in their symptoms. One provider said,

*“You have to explain to them that it took a long time to get there and it can take sometimes longer to get you back to where you were….And that’s one thing that we’ll discuss with them. Within a reasonable amount of time, within a couple of months or so, or sometimes even a couple weeks, you should experience some kind of change.”* – Chiropractor 8, Seattle

Providers differed in their strategies for communicating information regarding timeframes for treatment and progress. Some providers indicated that they typically recommend a treatment plan of a certain number of visits over a number of weeks before assessing progress. Other providers said that there is too much individual variability among patients to generalize a timeframe for progress and they therefore tailor treatment recommendations according to their assessment of each individual patient.

Providers, with the exception of yoga instructors, also explicitly manage patient expectations with regard to effects after the first treatment. Providers said that patients often come to them with the expectation that they will experience immediate pain relief after treatment. Although such relief is common, patients who do not experience such results may be disappointed and discontinue treatment early on. Providers said that they typically try to diffuse those expectations by outlining more realistic timeframes. One chiropractor said,

*“Yeah, I do [try to manage expectations], because I hate people that go in thinking that it’s only going to take one adjustment and if it doesn’t, they’ll think it was my fault. And everybody responds so differently. So that’s the hardest thing to sit down with somebody and tell them, ‘OK, it’s going to take a few sessions to get you where you want to be.’” –* Chiropractor 9, Tucson

In addition, providers indicated that patients who experience an initial positive response often develop unrealistically high expectations for overall treatment effectiveness. Providers considered this problematic because this improvement often is not sustained. Providers said that it is important to acknowledge and inform patients about the range of possibilities up front to effectively manage the expectation of a “quick fix.” Otherwise, patients may express frustration and disappointment with the treatment. According to one provider,

*“I try to do a lot of education because oftentimes what happens is they feel great after one session. It’s amazing, it’s the cure. And then after a couple days, they start to feel their symptoms again. And they’re really surprised by that. So then they think, ‘Oh no, this didn’t work after all,’ So they get really disappointed and so I try to, especially right after the first session before they leave if I think there’s any chance there may be any side effects, I talk to them about that and drinking water, but also about that you may feel better and it may not last.” –* Massage therapist 10, Seattle

Another provider said,

*“Sometimes I’m really surprised when somebody’s all better after one treatment. And I’m kind of a little bit nervous about that because that’s an expectation thing because I’ve really learned, like I’ve always been excited when people are better, of course, but their expectations can be really hard to manage after that. If it seems like a miracle and after one treatment everything’s gone, and if it comes back in two days and then they say it doesn’t work, that can be tricky to manage and that’s why I try to educate them in the beginning, that if you have any improvement after this treatment, even if it’s just for an hour or a day or just a little bit of improvement, I want you to know that’s a sign we’re heading in the right direction.” –* Acupuncturist 11, Seattle

#### *Treatment experience expectations*

CAM providers said they manage patients’ expectations with regard to a number of components of the treatment itself, including what it would consist of, how it would feel, and circumstances in which treatment is appropriate. Different types of providers cited unique reasons for managing expectations around treatment experience, particularly among patients new to their type of treatment.

Chiropractors and acupuncturists often said that patients who were naïve to their type of treatment frequently expressed anxiety and fear about the treatment and if it will be painful. These providers said that they walk new patients through the examination and treatment, explaining in detail what they were going to do and how it might feel. For example, one acupuncturist explained,

*“I do my best when [the patent] first comes in to explain to them what to expect…when they are on the bed and they’re ready for me to put the needles in…so that they’re not too scared. I say ‘First, I’m going to put the needles in,’ and I put several in from head to toe and I’m not going to talk much while I do it because the intention behind my needling is very important for getting results. And I tell them what I want [them] to do because everyone wonders if it hurts. That’s one of the bigger questions.”* – Acupuncturist 11, Seattle

Providers said being clear, thorough and transparent put the patient at ease and helped establish trust and rapport. There was also a belief that this approach improved outcomes because the treatment is more effective when the patient is relaxed. When discussing changes in expectations, one chiropractor said,

*“I think it becomes easier for them to accept the treatment. And when they become more comfortable with the treatment and because they know what to expect when they come in the office…your relationship gets better and better and it gets easier and easier to treat the problem.”* – Chiropractor 8, Seattle

Massage therapists and yoga instructors discussed correcting patients’ existing notions of massage or yoga therapy. They indicated that patients often bring a potentially harmful “no pain, no gain” mentality to treatment. For example, massage patients might ask the provider to “go deeper” and yoga students might push their bodies into poses that are painful. One yoga instructor said,

*“I will see it [their expectation] in their body. They will not be saying anything and I will say, ‘Did that feel okay?’ And they’ll go, ‘No!’… And it’s just so often having to bring a person back to where they are right now….what I’m hearing is you’re talking to me about how bad you want to be over here but you’re right here. Can we bring you back to somewhere where you can get interested? Can you get interested in this thing that’s going on for you right now … Because if you can get interested and stay put with it, then your expectations can be more reasonable.”* – Yoga instructor 12, Seattle

Providers said they manage these expectations through patient education and by explaining that the treatment may be more effective with a less aggressive approach or with a focus on an area other than where the pain is located. For example, yoga instructors may spend a lot of focusing on breathing before moving on to more challenging movements and massage therapists may work on the hamstrings or the abdomen as part of a treatment for chronic low back pain.

## Discussion

Our study provides new insights into the construct of “patient expectations of treatment” from the perspective of CAM providers. We found that chiropractors, acupuncturists, massage therapists, and yoga instructors all acknowledged the importance of effectively managing several aspects of expectations of patients with chronic low back pain throughout the treatment process. These include the roles and responsibilities of providers and patients, treatment outcomes, timeframe for improvement, and treatment experience. These findings indicate that CAM providers view patient expectations as complex and dynamic and that they play an active role responding to and shaping these expectations throughout the treatment process.

Findings from prior studies of the relationship between pre-treatment expectations and outcomes of care for both conventional medical and CAM therapies have been inconsistent [11-20]. Some clinical trials have suggested that pretreatment expectations are associated positively with CAM therapy outcomes. Although previous research suggests that CAM providers attempt to increase patient expectations of positive outcomes [11-15], CAM providers we interviewed said they attempt to ensure that their patients’ expectations of the treatment process and likely outcomes are realistic. CAM providers characterized this approach as “patient education” and reported that it helps sustain patient engagement in treatment and reduces the risk of patient dissatisfaction with the treatment process and outcomes. Management of patient expectations is not unique to CAM providers and has been reported to occur in conventional health care settings [29,36].

Our finding of changes in patient expectations over time are consistent with previous research [16,17] and suggest expectations are influenced by various factors including treatment experience and CAM providers’ deliberate efforts to provide education and manage expectations throughout treatment. Providers reported that patients’ expectations become more realistic as they become more familiar with the therapy and its effects. Their behavior is consistent with Caspi’s (2003) assertion that a good clinician will reframe a patient’s health concerns in ways to engender hope and positivity within a realistic and open perspective on the value of the therapy [37].

We found that providers said they often educated patients regarding their own roles in the healing process. At least some of the education, focused on the role of self-care, is likely to overlap with that of conventional health care providers.

However, the lifestyle and self-responsibility recommendations of CAM providers may differ somewhat from those of conventional providers.

Our study did not examine how patients interpret or perceive providers’ attempts to manage expectations. The extent to which patient expectations are informed by patients’ experience with CAM treatment as a whole (including treatment outcomes, interaction with CAM providers, etc.) versus other factors (e.g., societal and cultural beliefs regarding CAM, friends’ or family members’ experiences with CAM) remains unknown.

### Implications for clinical research

CAM providers view patient expectations regarding treatment as dynamic over time and influenced by both providers’ purposeful attempts to educate patients and patients’ experiences with the therapy. Assessment of patient expectations only before patients begin a new treatment is likely to miss potentially important changes in expectations that often occur once treatment is initiated and during the course of therapy. This area of research is challenging, however, because of the likely reciprocal relationships between expectations and outcomes over the course of therapy. Further research is needed to better understand the extent to which patient expectations at various points in time over the course of receiving a therapy are associated with patient outcomes, and the causal relationships between expectations and outcomes. Further research is also needed to better understand the mechanisms and dynamics of any relationships found between expectations and outcomes.

### Limitations

These findings are based on interviews with a purposeful sample of providers in two geographic areas and reflect their self-reported perspectives and understanding of patient expectations. The extent to which these findings may generalize to other CAM providers in other settings is unknown. However, although the context for CAM providers differed between Seattle and Tucson (e.g., differences in licensure requirements and insurance coverage for CAM therapies), similar themes emerged. Finally, the study did not capture patient perspectives or their responses to providers’ efforts to manage their expectations.

## Conclusion

This study begins to elucidate the complex issue of patient expectations from the perspectives of CAM providers. We found that CAM providers of various types think expectations are an important component of patient experience with CAM therapy and acknowledge using a variety of patient education approaches to manage expectations of patients with chronic low back pain. CAM providers noted the importance of ensuring that patient expectations are realistic from the provider’s perspective, and acknowledged that this often meant attempting to adjust patients’ expectations downward.

## Abbreviations

CAM: Complementary and alternative medicine.

## Competing interests

The authors declare they have no competing interests.

## Authors’ contributions

LS participated in interview guide development, interviewed providers, coded and analyzed the data, and drafted the manuscript. CH led interview guide development, interviewed providers, coded and analyzed the data and helped to draft the manuscript. EE participated in interview guide development, recruited and interviewed providers, coded and analyzed the data, and reviewed and revised the manuscript. CR contributed to the design of the study, participated in interview guide development, recruited providers, reviewed transcripts and helped interpret the data, and reviewed and revised the manuscript. JT contributed to the literature review, participated in interview guide development, reviewed transcripts and helped interpret the data, and reviewed and revised the manuscript. DC contributed to the study design, participated in interview guide development, reviewed transcripts and helped interpret the data, and reviewed and revised the manuscript. CS participated in interview guide development, recruited providers, and reviewed transcripts and helped interpret the data. KS is the Principal Investigator; she conceived of and designed the study, contributed to the literature review, participated in interview guide development, recruited providers, reviewed transcripts and helped interpret the data, and reviewed and revised the manuscript. All authors read and approved the final manuscript.

## Pre-publication history

The pre-publication history for this paper can be accessed here:

http://www.biomedcentral.com/1472-6882/12/234/prepub

## Supplementary Material

Additional file 1 Conceptual model of factors influencing patient expectations. Click here for file

## References

[B1] Di Blasi Z Harkness E Ernst E Georgiou A Kleijnen J Influence of context effects on health outcomes: a systematic review Lancet 2001 357 757 762 10.1016/S0140-6736(00)04169-6 11253970

[B2] Thomas KB General practice consultations: is there any point in being positive? Br Med J (Clin Res Ed) 1987 294 1200 1202 10.1136/bmj.294.6581.1200 PMC1246362 3109581

[B3] Main CJ Foster N Buchbinder R How important are back pain beliefs and expectations for satisfactory recovery from back pain? Best Pract Res Clin Rheumatol 2010 2 205 217 20227642 10.1016/j.berh.2009.12.012

[B4] Crow R Gage H Hampson S Hart J Kimber A Thomas H The role of expectancies in the placebo effect and their use in the delivery of health care: a systematic review Health Technol Assess 1999 3 1 96 10448203

[B5] Turner JA Deyo RA Loeser JD Von Korff M Fordyce WE The importance of placebo effects in pain treatment and research JAMA 1994 271 1609 1614 10.1001/jama.1994.03510440069036 7880221

[B6] Bausell RB Snake Oil Science 2007 New York: Oxford University Press

[B7] Ernst E Acupuncture–a critical analysis J Intern Med 2006 259 125 137 10.1111/j.1365-2796.2005.01584.x 16420542

[B8] Lynöe N Is the effect of alternative medical treatment only a placebo effect? Scan J Soc Med 1990 18 149 153 10.1177/140349489001800210 2195649

[B9] Beyerstein BL Alternative medicine and common errors of reasoning Acad Med 2001 76 230 237 10.1097/00001888-200103000-00009 11242572

[B10] Shang A Huwiler-Muntener K Nartey L Juni P Dorig S Sterne JA Pewsner D Egger M Are the clinical effects of homoeopathy placebo effects? Comparative study of placebo-controlled trials of homoeopathy and allopathy Lancet 2005 366 726 732 10.1016/S0140-6736(05)67177-2 16125589

[B11] Linde K Witt CM Streng A Weidenhammer W Wagenpfeil S Brinkhaus B The impact of patient expectations on outcomes in four randomized controlled trials of acupuncture in patients with chronic pain Pain 2007 12 247 270 10.1016/j.pain.2006.12.006 17257756

[B12] Myers SS Phillips RS Davis RB Cherkin DC Legedza A Kaptchuk TJ Patient expectations as predictors of outcome in patients with acute low back pain J Gen Intern Med 2008 23 148 153 10.1007/s11606-007-0460-5 18066631 PMC2359167

[B13] Lamb SE Lall R Hansen Z Withers EJ Griffiths FE Szczepura A Design considerations in a clinical trial of a cognitive behavioural intervention for the management of low back pain in primary care: back skills training trial BMC Musculoskelet Disord 2007 8 14 10.1186/1471-2474-8-14 17316434 PMC2147057

[B14] Goldstein MS Morgenstern H Hurwitz EL Yu F The impact of treatment confidence on pain and related disability among patients with low-back pain: results from the University of California, Los Angeles, low-back pain study Spine J 2002 2 391 399 discussion 9-401 10.1016/S1529-9430(02)00414-X 14589256

[B15] Kalauokalani D Cherkin DC Sherman KJ Koepsell TD Deyo RA Lessons from a trial of acupuncture and massage for low back pain: patient expectations and treatment effects Spine 2001 26 1418 1424 10.1097/00007632-200107010-00005 11458142

[B16] Sherman KJ Cherkin DC Ichikawa L Avins AL Delaney K Barlow WE Khalsa PS Deyo RA Treatment expectations and preferences as predictors of outcome of acupuncture for chronic back pain Spine 2010 35 1471 1477 20535051 10.1097/BRS.0b013e3181c2a8d3 PMC2895682

[B17] Thomas KJ MacPherson H Thorpe L Brazier J Fitter M Campbell MJ Randomized controlled trial of a short course of traditional acupuncture compared with usual care for persistent non-specific low back pain BMJ 2006 333 623 10.1136/bmj.38878.907361.7C 16980316 PMC1570824

[B18] Galer BS Schwartz L Turner JA Do patient and physician expectations predict response to pain-relieving procedures? Clin J Pain 1997 13 348 351 10.1097/00002508-199712000-00013 9430816

[B19] Smeets RJ Beelen S Goossens ME Schouten EG Knottnerus JA Vlaeyen JW Treatment expectancy and credibility are associated with the outcome of both physical and cognitive-behavioral treatment in chronic low back pain Clin J Pain 2008 24 305 315 10.1097/AJP.0b013e318164aa75 18427229

[B20] Smeets R Active Rehabilitation for Chronic Low Back Pain: Cognitive-behavioral, Physical or Both? 2006 Maastricht: Maastricht University 10.1186/1471-2474-7-5 PMC1382224 16426449

[B21] Feinstein AR Post-therapeutic response and therapeutic “style”: re-formulating the “placebo effect. J Clin Epidemiol 2002 5 427 429 12007543 10.1016/s0895-4356(01)00495-4

[B22] Pusic AL Klassen AF Snell L Cano SJ McCarthy C Scott A Cemal Y Rubin LR Cordeiro PG Measuring and managing patient expectations for breast reconstruction: impact on quality of life and patient satisfaction Expert Rev Pharmacoecon Outcomes Res 2012 2 149 158 22458616 10.1586/erp.11.105 PMC4182909

[B23] Bekker HL Luther F Buchanan H Developments in making patients’ orthodontic choices better J Orthod 2010 3 217 224 20805351 10.1179/14653121043119

[B24] Dmochowski RR Newman DK Impact of overactive bladder on women in the United States: results of a national survey Curr Med Res Opin 2007 23 65 76 10.1185/030079907X159533 17257467

[B25] Ralston JD Revere D Robins LS Goldberg HI Patients’ experience with a diabetes support programme based on an interactive electronic medical record: qualitative study BMJ 2004 328 1159 1162 10.1136/bmj.328.7449.1159 15142919 PMC411089

[B26] Doering LV McGuire AW Rourke D Recovering from cardiac surgery: what patients want you to know Am J Crit Care 2002 11 333 343 12102434

[B27] Crabtree TD Puri V Bell JM Bontumasi N Patterson GA Kreisel D Krupnick AS Meyers BF Outcomes and perception of lung surgery with implementation of a patient video education module: a prospective cohort study J Am Coll Surg 2012 214 816 821 e2 10.1016/j.jamcollsurg.2012.01.047 22464659

[B28] Kearney M Jennrich MK Lyons S Robinson R Berger B Effects of preoperative education on patient outcomes after joint replacement surgery Orthop Nurs 2011 30 391 396 10.1097/NOR.0b013e31823710ea 22124192

[B29] Shuldham C A review of the impact of pre-operative education on recovery from surgery Int J Nurs Stud 1999 36 171 177 10.1016/S0020-7489(99)00010-3 10376227

[B30] Stone DA Kerr C Jacobson E Conboy LA Kaptchuk TJ Patient expectations in placebo-controlled randomized clinical trials J Eval Clin Pract 2004 11 77 84 15660541 10.1111/j.1365-2753.2004.00512.x

[B31] Bishop MD Bialosky JE Cleland JA Patient expectations of benefit from common interventions for low back pain and effects on outcome: secondary analysis of a clinical trial of manual therapy interventions J Man Manip Ther 2011 19 20 25 10.1179/106698110X12804993426929 22294850 PMC3172953

[B32] Barnes PM Bloom B Nahin R Complementary and Alternative Medicine Use Among Adults and Children 2008 United States National Health Statistics Report 12 19361005

[B33] Chou R Qaseem A Snow V Casey D Cross JT Jr Shekelle P Owens DK Diagnosis and treatment of low back pain: a joint clinical practice guideline from the American College of Physicians and the American Pain Society Ann Intern Med 2007 147 478 491 17909209 10.7326/0003-4819-147-7-200710020-00006

[B34] Santaguida PL Gross A Busse J Gagnier J Walker K Bhandari M Raina P Complementary and alternative medicine in back pain utilization report Evid Rep Technol Assess (Full Report) 2009 177 1 221 PMC4781194 20629474

[B35] Borkan J Crabtree B, Miller W Immersion/crystallization Doing qualitative research 1999 2 Thousand Oaks: Sage Publications 179 194

[B36] Greene KA Harwin SF Maximizing patient satisfaction and functional results J Knee Surg 2011 24 19 24 10.1055/s-0031-1275389 21618934

[B37] Caspi O Rakel D Activating the Healing Response Integrative Medicine 2003 Philadelphia: Saunders 703 710

