# Supplementary material for: Complementary and alternative medicine (CAM) providers’ views of chronic low back pain patients’ expectations of CAM therapies: a qualitative study
Source: BMC Complement Altern Med. 2012 Nov 27;12:234. doi: 10.1186/1472-6882-12-234 (PMC3533863; doi:10.1186/1472-6882-12-234)
Supplement: Additional file 1 — Conceptual model of factors influencing patient expectations. [file 1472-6882-12-234-S1.doc]

Conceptual Model of Factors Influencing Patient Expectations
